# Feasibility of HEMS performed prehospital extracorporeal-cardiopulmonary resuscitation in paediatric cardiac arrests; two case reports

**DOI:** 10.1186/s13049-023-01119-4

**Published:** 2023-09-19

**Authors:** Lars Mommers, Cornelis Slagt, Freek Coumou RN, Ruben van der Crabben, Xavier Moors, Dinis Dos Reis Miranda

**Affiliations:** 1 Department of Anaesthesiology and Pain Medicine, Maastricht University Medical Centre, P.Debyelaan 25, Maastricht, 6229 HX The Netherlands; 2 Helicopter Emergency Medical Service Lifeliner 3 Radboudumc, Geert Grooteplein 10, Nijmegen, 6525 GA The Netherlands; 3 Department of Anaesthesiology, Pain and Palliative Medicine, Radboud University Medical Centre, Geert Grooteplein 10, Nijmegen, 6525 GA The Netherlands; 4 Department of Anaesthesiology, Erasmus University Medical Centre, Dr. Molewaterplein 40, Rotterdam, 3015 GD The Netherlands; 5 Helicopter Emergency Medical Service Lifeliner 2, Dr. Molewaterplein 40, Rotterdam, 3015 GD The Netherlands; 6 Department of Adult Intensive Care, Erasmus University Medical Centre, Dr. Molewaterplein 40, Rotterdam, 3015 GD The Netherlands

**Keywords:** Paediatric extracorporeal life support, Cardiopulmonary resuscitation, Out-of-hospital cardiac arrest, Case report

## Abstract

**Introduction:**

A broad range of pathophysiologic conditions can lead to cardiopulmonary arrest in children. Some of these children suffer from refractory cardiac arrest, not responding to basic and advanced life support. Extracorporeal-Cardiopulmonary Resuscitation (E-CPR) might be a life-saving option for this group. Currently this therapy is only performed in-hospital, often necessitating long transport times, thereby negatively impacting eligibility and chances of survival. We present the first two cases of prehospital E-CPR in children performed by regular Helicopter Emergency Medical Services (HEMS).

**Case presentations:**

The first patient was a previously healthy 7 year old boy who was feeling unwell for a couple of days due to influenza. His course deteriorated into a witnessed collapse. Direct bystander CPR and subsequent ambulance advanced life support was unsuccessful in establishing a perfusing rhythm. While doing chest compressions, the patient was seen moving both his arms and making spontaneous breathing efforts. Echocardiography however revealed a severe left ventricular impairment (near standstill). The second patient was a 15 year old girl, known with bronchial asthma and poor medication compliance. She suffered yet another asthmatic attack, so severe that she progressed into cardiac arrest in front of the attending ambulance and HEMS crews. Despite maximum bronchodilator therapy, intubation and the exclusion of tension pneumothoraxes and dynamic hyperinflation, no cardiac output was achieved.

**Intervention:**

After consultation with the nearest paediatric E-CPR facilities, both patients were on-scene cannulated by regular HEMS. The femoral artery and vein were cannulated (15-17Fr and 21Fr respectively) under direct ultrasound guidance using an out-of-plane Seldinger approach. Extracorporeal Life Support flow of 2.1 and 3.8 l/min was established in 20 and 16 min respectively (including preparation and cannulation). Both patients were transported uneventfully to the nearest paediatric intensive care with spontaneous breathing efforts and reactive pupils during transport.

**Conclusion:**

This case-series shows that a properly trained regular HEMS crew of only two health care professionals (doctor and flight nurse) can establish E-CPR on-scene in (older) children. Ambulance transport with ongoing CPR is challenging, even more so in children since transportation times tend to be longer compared to adults and automatic chest compression devices are often unsuitable and/or unapproved for children. Prehospital cannulation of susceptible E-CPR candidates has the potential to reduce low-flow time and offer E-CPR therapy to a wider group of children suffering refractory cardiac arrest.

## Introduction

A broad range of pathophysiologic conditions can lead to cardiopulmonary arrest in children. Resuscitation councils visualise the cardiac arrest care as a chain of survival beginning with early identification, adequate bystander Basic Life Support (BLS) including early defibrillation using an Automated External Defibrillator (AED), followed by Advanced Life Support (ALS) therapies to achieve spontaneous circulation and subsequent post-resuscitation care.

Extracorporeal-CardioPulmonary Resuscitation (E-CPR) might be an option for those children suffering refractory cardiac arrest. E-CPR is a technique in which the patient is connected to a miniaturized heart-lung machine via large bore cannulas in the common femoral artery and vein, thereby restoring the patient’s circulation and providing time for diagnosis and possible treatment. E-CPR is widely used for in-hospital cardiac arrest. Since (E-)CPR is a time-critical process, initiatives are being taken to perform E-CPR earlier in the resuscitation course and therefore also prehospitally. Randomized controlled trials showing benefit of prehospital E-CPR for adults are not yet available, however prehospital E-CPR seems feasible in adults and is gaining momentum [1–4]. There is however no program supporting prehospital E-CPR in children.

We present the first two cases of pre-hospital paediatric E-CPR conducted by two regular helicopter emergency medical service (HEMS) consisting of small crews (pilot, physician and flight nurse). The cases are described according to the CARE guideline from the Equator network [5].

## Therapeutic intervention - paediatric pre-hospital cannulation

Several HEMS providers in the Netherlands are intensively trained to perform pre-hospital E-CPR cannulation. This training is part of the multi-centre “Onscene Trial” which started in October 2021. Prehospital cannulation is performed on highly selected patients by a two person Helicopter Emergency Medical Service (HEMS) crew (doctor and flight nurse). It is however possible to cannulate patients outside this trial with the right indications and after acceptance by the receiving E-CPR centre. General accepted criteria for paediatric E-CPR are an age above 7 years, witnessed arrest and/or signs of life, no-flow time < 5 min, adequate BLS, end-tidal CO_2_ > 1.3 kPa (10 mmHg) and a visible femoral artery and vein on ultrasound.

The procedure is performed under direct ultrasound guidance, using an out-of-plane technique for constant vessel orientation given the possible shifting from ongoing chest compressions. This approach also facilitates puncture of both vessels at the most anterior aspect (i.e. ‘on top’) since lateral or medial puncture might challenge cannulation and vascular patch repair afterwards. After both guidewires are introduced, a mandatory confirmation (by doctor and flight nurse) is performed visualizing two wires in two different vessels (i.e. artery and vein). Subsequent dilatation is performed to accommodate a 15-17Fr arterial and 21Fr venous cannula. Connection to the CardioHelp HLS 7.0 set (Getinge, Maquet Cardiopulmonary GmbH, Germany) is made using a wet-to-wet connection. An intra-arterial catheter (20G) is inserted also under ultrasound guidance in the contralateral femoral artery and connected to a direct mean arterial pressure (MAP) transducer (Compass, Medline, Warrington, UK).

## Patient information

### Case 1

A previously healthy 7 year-old boy, 25 kg, is feeling unwell for 5 days. Diagnosed with influenza by his general practitioner, his clinical course deteriorates into a witnessed collapse. Caregivers directly called emergency services and started immediate bystander CPR. Ambulance and HEMS were simultaneously deployed. Initially, the AED had given no shocks, however later in the course a single episode of ventricular fibrillation was seen for which defibrillation was performed. Intravenous access was established for epinephrine and fluid bolus administration and ventilation was performed through bag-valve mask. After three blocks of ALS, end-tidal CO_2_ rose to 4.2 kPa and return of circulation was confirmed. The supraventricular rhythm of 90/min however progressed towards near asystole within minutes for which BLS and ALS were reinstated. While doing chest compressions the patient was seen moving both arms and making spontaneous breathing efforts. Point-of-care ultrasound (POCUS) revealed a near cardiac standstill of the left ventricle and minimal contractility of the right ventricle. The nearest paediatric intensive care unit (PICU) was consulted to discuss acceptance for E-CPR. Thirty-seven minutes after the initial collapse, approval was received to cannulate this patient on-scene. The femoral artery and vein were cannulated (15Fr and 21Fr respectively) as previously described and proper ECLS flow (2.1 l/min) was established in 20 min (5 min of preparation and 15 min of cannulation), 52 min after the initial collapse. Initial MAP measurement was 70 mmHg. The patient was packed and transported uneventfully to the PICU with spontaneous breathing efforts and reactive pupils during transport.

### Case 2

A 15 year old girl, known with bronchial asthma and poor medication compliance was in a status asthmaticus. Ambulance and HEMS were simultaneously deployed. On arrival of the paramedics she was unconscious but with a pulse. On arrival of the HEMS team, she was poorly circulated, hypoxemic unconscious but with spontaneous movements. Auscultation revealed no incoming breath sounds. After salbutamol per inhalation and intravenous administration, breath sounds became audible. She suffered a severe bradycardia deteriorating into asystole shortly after. ALS was immediately instituted. Her initial expiratory CO_2_ was > 5.0 kPa after intubation (without a plateau phase) with high pressures on bag-valve ventilation. Tube disconnection and thoracic pressure revealed no dynamic hyperinflation; pneumothoraxes were excluded. The nearest E-CPR centre was consulted to gain approval for E-CPR. The femoral artery and vein were cannulated (17Fr and 21Fr respectively) as previously described and proper ECLS flow (3.8 l/min) was established in 16 min (6 min of preparation and 10 min of cannulation), 26 min after the collapse. The initial MAP was 100 mmHg. Within a minute after restarting ECLS flow, the rhythm converted to a sinus rhythm. The patient was packed and transported uneventfully to the PICU with spontaneous breathing efforts and reactive pupils during transport.

## Timeline

**Fig. 1:**
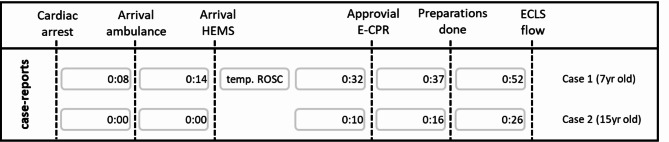
Timescale E-CPR interventions both case-reports **Legenda**: the cardiac arrest represents the start (0:00), important events are given until ECLS flow is established **Abbreviations**: HEMS = Helicopter Emergency Medical Service, E-CPR = Extracorporeal-CardioPulmonary Resuscitation, ECLS = ExtraCorporeal Life Support, (temp) ROSC = (temporary) Return of Spontaneous Circulation

Timelines of both pre-hospital E-CPR procedures are displayed in Fig. 1.

## Follow-up and outcomes

Patient 1 showed severe left ventricle impairment in the emergency department and a positive test for influenza B. With additional inotropes and ECLS flow of 2 l/min, opening of the aortic valve could be achieved. Empiric antibiotics were instituted directly. Despite initial metabolic improvement, the patient developed a progressive distributive shock hindering ECLS flow. A second venous cannula could not improve ECLS flow and therapy was withdrawn the day after. Additional blood cultures and genetic testing revealed no abnormalities, leaving a severe influenza B infection with accompanying myocarditis as the diagnosis.

Patient 2 arrived at the hospital with a blood pressure of 117/80 and pressure controlled ventilated with peak inspiratory pressures of 30 mbar and a PEEP of 10 mbar which resulted in a ventilation of 25 × 3.5 ml/kg. At ICU arrival, here pupils deteriorated, became wide and non-reactive to light. The electroencephalogram was and remained isoelectric. Treatment was withdrawn two days after the arrest.

## Discussion

Delays in resuscitation are important as survival (in children) drops significantly over time [6]. Although there is no uniform time definition of ‘refractory’ cardiac arrest in children, a timeframe of 10–30 min is often cited in adults together with the advice to limit low-flow times to under 60 min for E-CPR procedures [3, 7–13]. Looking at the broad overall group of paediatric E-CPR survival to hospital discharge, survival ranges between 30 and 50%.[6, 14–19].

**Fig. 2:**
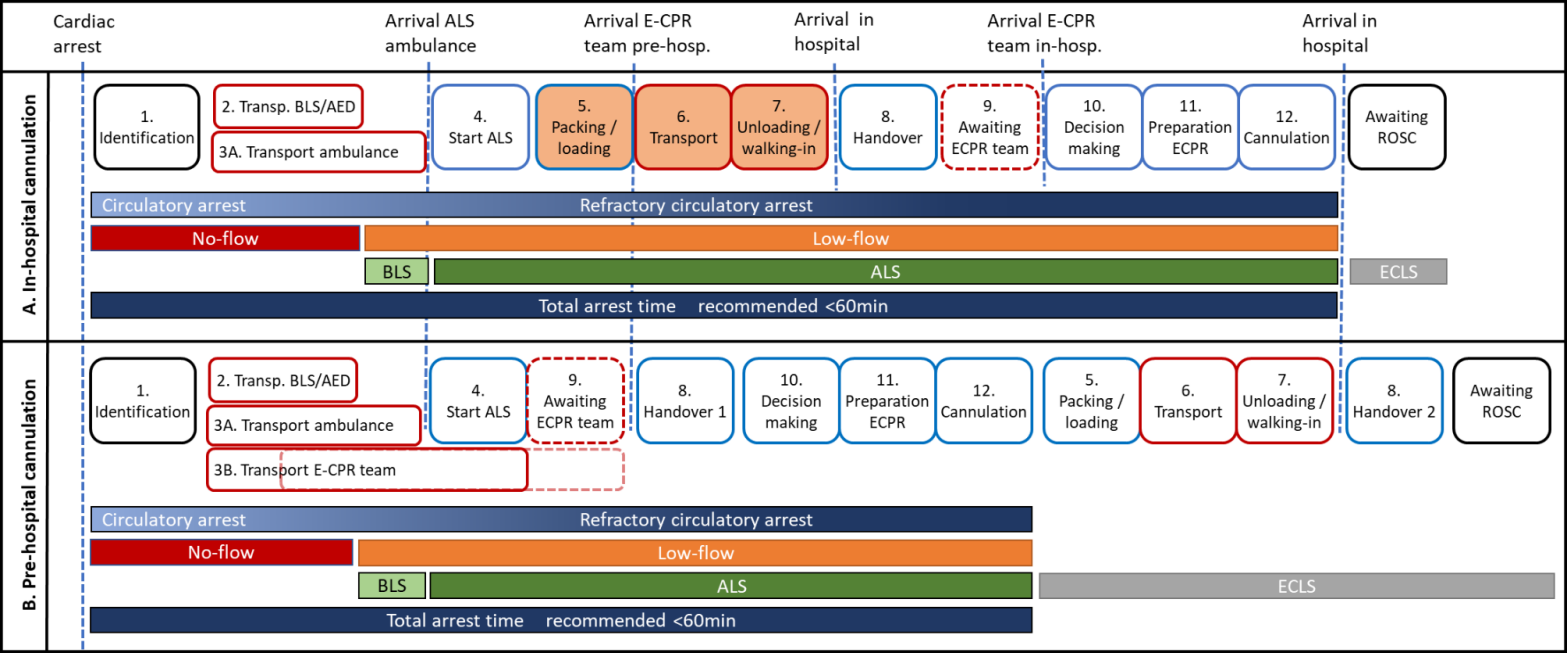
Visualising time consuming variables in (E-)CPR **Legenda**: time-consuming variables in (E-)CPR with in-hospital cannulation (**A**) versus pre-hospital cannulation (**B**) as well as the timeframes of both case reports (**C**) Blue delineated components can be mitigated by proper training, red delineated components are rather fixed. The optional component 9 (dotted red line) is depending on E-CPR activation method and the geographic distance to be covered. The orange shaded components (A 5–7) poses a particular challenge with CPR in-transit in resource limited environment, especially in children. These variables are omitted from low-flow time in case of pre-hospital cannulation **Abbreviations**: BLS = Basic Life Support, AED = Automated External Defibrillator, ALS = Advanced Life Support, E-CPR = Extracorporeal-CardioPulmonary Resuscitation, ECLS = ExtraCorporeal Life Support, ROSC = Return of Spontaneous Circulation

Since paediatric E-CPR centres are less widespread compared to adult E-CPR centres, transport times tend to be longer, hereby negatively affecting low-flow times. In addition, transport with ongoing paediatric ALS care is more challenging compared to adult ALS since available staff is limited and automatic compression devices are not always available and/or approved for children [3]. Most paediatric E-CPR centres subsequently limit their programme to in-hospital cardiac arrests. Both cases illustrate that pre-hospital E-CPR cannulation by properly trained HEMS is feasible. This approach might be beneficial to limit low-flow times. Previous publications have highlighted differences between pre-hospital and in-hospital cannulation [11, 20]. Fig. 2 shows a graphic composition of all time-consuming variables in (E-)CPR, differentiating modifiable variables that can be improved e.g. by intense training (blue delineated) versus relatively fixed variables (red delineated), e.g. due to geographical conditions. The orange shaded variables poses a particular challenge with ongoing CPR in-transit (in case of in-hospital cannulation) and can be omitted with on-scene cannulation.

Data on prehospital paediatric E-CPR for comparison with these case-reports is lacking. Cannulation is anticipated to be more challenging since vessel diameters are smaller. In adults, the vascular complication rate of E-CPR in cardiogenic shock and cardiac arrest patients is 15–30% [21, 22]. More specific, access site complications during in-hospital E-CPR for OHCA patients may occur in up to 20% [23] with cannulation failure occurring in 15% when the procedure is performed by trained intensivists [24]. Diameter of the femoral artery tends to be an independent risk factor for cannulation failure, with an optimal threshold diameter of 7.5 mm [24]. Also in the prehospital setting, E-CPR by dedicated teams seems feasible and safe in adults [1, 2]. Large studies investigating the safety and feasibility of general HEMS teams performing prehospital adult E-CPR are still ongoing [25, 26].

Another backdraft in prehospital paediatric E-CPR might be the emotional burden on (H)EMS crew. After debriefing both cases, no excessive emotional distress was mentioned by the (H)EMS crew members.

Given the costs, labour intensiveness and possible complications of E-CPR, indications have to be weighted carefully. Given the signs of life and short low-flow intervals at that time, both patients would have been accepted for in-hospital E-CPR (had they been in the ED at that time) [6, 13]. The unfortunate outcomes of both patients however stresses the utmost important question: ‘Who should we cannulate?’ This case-series will not provide an answer, but hopefully it can contribute to more research within the paediatric E-CPR community as it did within the adult population.

## Conclusions

Prehospital E-CPR cannulation of (older) children by regular HEMS crews seems feasible with short cannulation times. Having this intervention done by regular HEMS can reduce low-flow times and provide a potential wider population access to this rescue therapy. More research is needed to establish the safety and the benefits of this intervention in the paediatric population.

## Data Availability

Not applicable.

## List of abbreviations

AED: Automated External Defibrillator
ALS: Advanced Life Support
BLS: Basic Life Support
(E-)CPR: (Extracorporeal-)Cardiopulmonary Resuscitation
ECLS: Extracorporeal Life Support
(H)EMS: (Helicopter) Emergency Medical Service
MAP: Mean Arterial Pressure
PICU: Paediatric Intensive Care Unit
POCUS: Point-of-Care Ultrasound
ROSC: Return of Spontaneous Circulation
